# Modern Medico-Psychology and Psychiatry

**Published:** 1895-07-27

**Authors:** T. S. Clouston

**Affiliations:** Lecturer on Mental Diseases, Edinburgh University, and Physician Superintendent Royal Asylum, Morningside


### Modern Medico-Psychology and Psychiatry.

By T. S. Clouston, M.D., Lecturer on Mental Diseases, Edinburgh University, and Physician Superintendent Royal Asylum, Morningside.

### PARTIAL ARRESTS AND POSTPONEMENTS OF BRAIN DEVELOPMENT.

I lately saw a boy, who was sharp and intelligent in a great many respects, who took about an average place at school, yet who from the time he was three years of age appropriated all sorts of property that was not his own, and could never be taught the difference between *meum* and *tuum*. He was utterly untruthful too. He stole all sorts of things when he had plenty of money in his pocket, and sometimes things he had no use for at the time. Money that was obtainable he could never resist trying to get hold of. He would tell most plausible stories as to how he obtained stolen articles, and would sometimes, when found out, express sorrow and beg that he should not be allowed to be in circumstances where he would be tempted again, averring most piteously that he could not help stealing and lying. He had been brought up carefully, was rich, and every pains had been taken to teach and create in him feelings of honour and morality. But he came of a neurotic stock, his father having committed suicide and there being insanity on his mother's side as well, and he himself was in body not well developed for his age. It is a terrible problem what to do with such a man as he grows up. There is no mental or moral, and almost no physical, defects that may not be accounted for on the theory of arrested development. It must not be understood, of course, that all or most moral defects are due to such a cause. Heredity, environment, education, example, principle, and the subtle but most powerful "atmosphere" in which persons are brought up, all contribute to moral strength and weakness, but all these must have a basis in a sound brain. The most important and the most difficult of all the developmental arrests are not those affecting the outward form of the body, or the visible shape of the organs, but are those affecting the self-nourishing power and energising of the brain cells through which mind and will and character are manifested. When one nerve centre in the brain lacks normal correlation to other centres, and when there is no organic solidarity in the different parts of the brain to work harmoniously towards the ends of organic and mental life, then we may have all kinds of apparently inexplicable conduct. The beginnings of the insane diathesis, and of the German "Paranoia," commonly make their appearance during the period of development. Lads and maidens who are very impracticable, highly unconventional, much too impulsive, or even too markedly "old-fashioned," should be carefully watched. There may be a sort of pathological stupidity or lethargy, and none of the normal curiosity of youth; or there may be a strange want of social inclination at this the most sociable of all ages. The youth ceases to mix with his friends, or to play games, or he shuts himself up, or takes to some kind of unnatural study in excess; or the developmental defect may take the form of the child's lack of self-control, continued into adolescence, or there may be an utter incompatibility of temper with father and mother, with friends and with masters. Enemies are causelessly made everywhere; or a frothy foolish sentimental religionism, with no steadying effect on the conduct, is developed; or we have sudden immoralities contrary to the tenour of the former life. I often see girls and youths who, up to fifteen or so, had been as ordinary persons in conduct, but who after that became the despair of teachers and parents, and the family skeletons at home. Clever enough intellectually, sometimes unusually so, and not grossly immoral, they are yet perverse, contradictory, outrageously unconventional, and utterly wanting in duty. In some of them egotism seems to become their only motive of conduct; all their affection and goodwill are concentrated on animals, tramps, and oddities; all their sympathy and good deeds flow towards the bad and the low; their "love affairs" are either absurd or entirely absent; their uncharity towards certain persons is unbounded, while towards others their charity is pugnaciously expressed, in both cases being educed on opposite principles from that of average good men and women. Often attempts at suicide are made from most inadequate causes. It is fortunate if such persons "make up" their postponed development between twenty and twenty-five; a few of them do so, and turn out useful members of society, with a rare genius among them once in a half century. Who would have thought that philosophers like Mill and Hume would have seriously contemplated suicide during their adolescence? A few emerge into a lurid light afterwards, as murderers and criminals like Guiteau. A few do much evil and some good, like King Louis II. of Bavaria, who, amidst all his preposterous eccentricities, was a steady patron of Wagner, and unhesitatingly threw Bavaria into the German scale against France in 1870, and yet ended by killing himself and his benevolent physician Von Gudden.

While we know that the same kind of nervous complaints and developmental arrests are apt to occur as hereditary manifestations in the same family, hare-lip following hare-lip, epilepsy producing epilepsy, insanity begetting insanity; yet, on the other hand, there is an hereditary interchangeability in all nervous affections, melancholia in grandparents, developmental arrests in the next generation, and more marked insanity and idiocy in the third.

All these examples of unrelational arrests and developments are neurotic in constitution, have a bad heredity, and some end by becoming insane. The facts and laws of the hereditary transmission of nervous mental diseases are as yet most obscure. I have had proof of mental disease passing over three generations of healthy people, and reappearing in exactly the same form in the fourth. We never know whether arrested development, insanity, epilepsy, or consumption will break out as the result of bad nervous heredity. Very many of them die of nervous ailments or consumption. When we consider the matter, we see that they are all only more marked examples of the unequal and irregular developments of body and mind which are almost universal among humanity in lesser degree.

